# Supplementary material for: In Vitro Characterization and Safety Assessment of Streptococcus salivarius, Levilactobacillus brevis and Pediococcus pentosaceus Isolated from the Small Intestine of Broiler Breeders
Source: Microorganisms. 2025 May 27;13(6):1231. doi: 10.3390/microorganisms13061231 (PMC12195246; doi:10.3390/microorganisms13061231)
Supplement: Supplementary file 1 [file microorganisms-13-01231-s001.zip › microorganisms-3594956-supplementary.pdf]

Supplementary Table S1: Antagonistic activity of LAB strains from the small intestine of broiler breeders.

Isolate ID	*L. monocytogenes*	*S. typhimurium*	*S. enteritidis*	*P. aeruginosa*	*S. aureus*
S. salivarius NKFS6	14,50 ±0,71	18,00 ±4,24	28,50 ±3,54	23,50 ±2,12	18,50 ±2,12
L. brevis NKFS7	20,50 ±0,71	17,50 ±0,71	29,00 ±1,41	21,50±0,71	23,00 ±4,24
L. brevis NKFS8	19,00 ±2,83	18,50 ±2,12	28,50 ±12,02	21,00 ±0,00	20,00 ±4,24
L. brevis NKFS9	25,00 ±7,07	21,00 ±1,41	30,00 ±0,00	24,50 ±0,71	19,50 ±4,95
P. pentosaceus NKFS10	23,00 ±4,24	23,00 ±7,07	13,50 ±0,71*	13,50 ±0,71	14,50 ±0,71*

P. pentosaceus NKFS11	19,00 ±0	15,50 ±3,54	13,00 ±1,41*	14,00 ±1,41	20,50 ±0,71
L. casei (ATCC 393)	21,00 ±1,41	25,50 ±2,12	27,00 ±4,24	17,50 ±3,54	27,00 ±2,82

Values are means ± SEM. Significant differences between the means of LAB isolates and the reference strain are indicated as * $p < 0.05$.
